# What improvements do general exercise training and traditional Chinese exercises have on knee osteoarthritis? A narrative review based on biological mechanisms and clinical efficacy

**DOI:** 10.3389/fmed.2024.1395375

**Published:** 2024-05-22

**Authors:** Xingbin Du, Rao Fan, Jianda Kong

**Affiliations:** ^1^Shandong Huayu University of Technology, Dezhou, China; ^2^Faculty of Education, Qufu Normal University, Qufu, China; ^3^College of Sports Science, Qufu Normal University, Qufu, China

**Keywords:** general exercise training, traditional Chinese exercise, knee osteoarthritis, biological mechanism, clinical efficacy

## Abstract

**Background:**

Knee osteoarthritis (KOA) is a disease that significantly affects the quality of life of patients, with a complex pathophysiology that includes degeneration of cartilage and subchondral bone, synovitis, and associations with mechanical load, inflammation, metabolic factors, hormonal changes, and aging.

**Objective:**

This article aims to comprehensively review the biological mechanisms and clinical effects of general exercise training and traditional Chinese exercises (such as Tai Chi and Qigong) on the treatment of KOA, providing references for the development of clinical exercise prescriptions.

**Methods:**

A systematic search of databases including PubMed, Web of Science, Google Scholar, and China National Knowledge Infrastructure (CNKI) was conducted, reviewing studies including randomized controlled trials (RCTs), observational studies, systematic reviews, and meta-analyses. Keywords included “knee osteoarthritis,” “exercise therapy,” “physical activity,” and “traditional Chinese exercise.”

**Results and conclusion:**

General exercise training positively affects KOA by mechanisms such as promoting blood circulation, improving the metabolism of inflammatory factors, enhancing the expression of anti-inflammatory cytokines, and reducing cartilage cell aging. Traditional Chinese exercises, like Tai Chi and Qigong, benefit the improvement of KOA symptoms and tissue repair by regulating immune function and alleviating joint inflammation. Clinical studies have shown that both types of exercise can improve physical function, quality of life, and pain relief in patients with KOA. Both general exercise training and traditional Chinese exercises are non-pharmacological treatment options for KOA that can effectively improve patients’ physiological function and quality of life. Future research should further explore the long-term effects and biological mechanisms of these exercise interventions and develop personalized exercise programs based on the specific needs of patients.

## 1 Introduction

Knee osteoarthritis (KOA), is a specific type of osteoarthritis (OA) that significantly affects patients’ quality of life (1). The pathogenesis of KOA involves multiple mechanisms, with the main pathological changes consisting of degeneration of cartilage and subchondral bone, as well as synovitis. The complexity of its etiology is attributed to the intricate relationships with factors such as mechanical overload, inflammation, metabolic factors, hormonal changes, and aging (2, 3).

In the early stages of KOA, alterations in the molecular composition and organization of extracellular matrix in articular cartilage cells lead to changes in water-binding capacity and decreased mechanical strength (cartilage softening), resulting in greater deformation under load (4). As the disease progresses, the damaged cartilage areas expand, involving not only the subchondral bone but also the joint capsule, ligaments, synovium, and periarticular muscles (5). There is even evidence suggesting that inflammation and fibrosis of the infrapatellar fat pad (IFP), also known as Hoffa’s fat pad, are closely associated with the development of KOA, and that the inflammation of IFP may lead to the production of various inflammatory mediators (such as cytokines and adipokines) within the knee joint, influencing its health and function (6, 7). Simultaneously, degenerative changes in the meniscus can lead to the development of KOA. Specifically, with increasing age, the meniscus gradually undergoes wear and tear, leading to instability of the knee joint and uneven loading of the articular surfaces, which accelerates the damage and wear of the articular cartilage and ultimately results in KOA (8). Additionally, articles have demonstrated that the interaction between the immune and nervous systems plays a crucial role in the initiation and maintenance of KOA pain (9, 10).

Currently, conventional treatment modalities such as drug therapy and surgical intervention have been proven to have some limitations (11, 12), whereas physical exercise has been shown to have a positive impact on KOA, particularly in terms of molecular biology, and exhibits potential to alleviate symptoms and improve the quality of life of patients (13). This suggests that exercise can be considered a non-pharmacological yet effective approach in the management of KOA. However, despite the numerous exercise regimens available, there is a lack of comprehensive reviews focusing on the biological mechanisms and clinical efficacy of general exercise training and traditional Chinese exercises for KOA.

Therefore, in this context, we have authored a narrative review aimed at providing a comprehensive overview of the benefits of general exercise training and traditional Chinese exercises on KOA, as well as exploring their respective biological mechanisms and clinical outcomes. The ultimate goal is to provide potential references for the development of clinical exercise prescriptions for KOA patients.

## 2 Materials and methods

### 2.1 Literature search

In conducting this narrative review, we systematically searched databases including PubMed, Web of Science, and Google Scholar using a combination of keywords such as “knee osteoarthritis,” “exercise therapy,” “physical activity,” “traditional Chinese exercises,” and “clinical outcomes.” In addition, as this review involves traditional Chinese exercises, we also searched for Chinese literature in the China National Knowledge Infrastructure (CNKI).

### 2.2 Inclusion and exclusion criteria

This review is guided by the following inclusion criteria: (i) types of studies: this includes randomized controlled trials (RCTs), observational studies, systematic reviews, meta-analyses, commentary articles, and expert opinions; (ii) subjects of the study: not limited to patients with KOA, the included studies may involve KOA patients, as well as healthy populations or individuals with other related conditions, in order to explore the general impact of exercise on knee joint health; (iii) interventions: the studies should involve interventions that include general exercise training (such as aerobic exercises, strength training, balance training, etc.) and/or traditional Chinese medicine exercises (such as Tai Chi, Qigong, Yi Jin Jing, etc.); (iv) study outcomes: studies should evaluate any outcomes related to KOA or knee joint health, including but not limited to pain relief, improvement in physical function, and enhancement of quality of life; (v) language of publication: there is no restriction on the language of publication, but studies with English full-text or abstracts are preferred.

Additionally, the following exclusion criteria are applied: (i) missing data: studies lacking key methodological details or with incomplete result data are excluded; (ii) studies of very low quality: studies that are of evidently low quality based on design, execution, result reporting, etc., and may affect the reliability of conclusions, are excluded; (iii) duplicate publications: for studies that have been published more than once, only the most comprehensive or final version of the manuscript is included.

## 3 Findings

### 3.1 KOA and its pathogenesis

KOA, characterized by joint degeneration, is a common orthopedic condition clinically manifesting as knee swelling, stiffness, and restricted mobility. In severe cases, it can lead to muscle atrophy or even disability, representing the most prevalent and impactful form of arthritis affecting the quality of life in middle-aged and elderly populations (14). The pathogenesis of KOA is highly complex, involving the interplay of multiple factors. Damage to the cartilaginous structure within the knee joint, influenced by age, genetics, and metabolic dysregulation, leads to an imbalance between the synthesis and degradation of proteoglycans. This, in turn, triggers cartilage degeneration (15).

Additionally, chronic inflammation represents another crucial pathogenic mechanism, with the affected joints exhibiting chronic low-grade inflammation. Inflammatory cells and mediators produced in this environment cause damage to the cartilage and joint structure (16). Synovial cells are a key factor in this inflammation, closely linking joint pain, inflammation, cartilage destruction, and synovial cell activity (17). Moreover, excessive mechanical loading on the knee joint is a contributing factor to KOA, with obesity, prolonged physical labor, and over-exercise being potential causes (18). To stabilize the injured joint, the body may increase the contact area between joint surfaces through bone formation, which, however, can restrict joint mobility (19).

Furthermore, the infrapatellar fat pad (IFP) has been recognized for its role in KOA as a source of cytokines/lipid mediators, which contribute to the observed inflammation and structural changes in OA (6, 7, 20). Research highlights pro-inflammatory cytokines secreted by IFP, such as IL-6 and TNFα, and adipokines like lipoproteins and leptin, which are associated with the pathophysiology of OA. IFP-derived fatty acids and their hydroxyl derivatives possess immunomodulatory properties, further linking IFP to OA inflammation (20). Additionally, recent studies have identified the significant roles of inflammatory mediators such as nerve growth factor (NGF), tumor necrosis factor (TNF), and interleukins (IL) 1 and 6 in the progression of KOA. These factors may exacerbate various pathological processes, including cartilage degeneration, bone formation, and joint overloading (21, 22).

### 3.2 Improvement mechanism of exercise on KOA: based on cartilage cell senescence

#### 3.2.1 General exercise training for the improvement of KOA: mainly based on cartilage cell senescence

Evidence has demonstrated that general exercise training can offset inflammatory factors, particularly generating positive effects on immunoregulation and anti-inflammatory mechanisms (23). This effect has been confirmed in cancer and is likely applicable to KOA through various mechanisms, including enhancing blood circulation, improving the metabolism of inflammatory factors, increasing the expression of anti-inflammatory cytokines, and reducing inflammation and chondrocyte senescence. Specifically, exercise training can prevent cartilage degradation, inhibit inflammation, prevent osteoporosis, improve joint function, and alleviate pain and stiffness. This includes various forms of exercise such as aerobic, strength training, neuromuscular exercises, balance training, and aquatic exercises. Studies on experimental animals have shown that these forms of exercise can reduce inflammation, delay the degeneration of cartilage and bones, and alter the structure of tendons and muscles (3).

**Figure 1:**
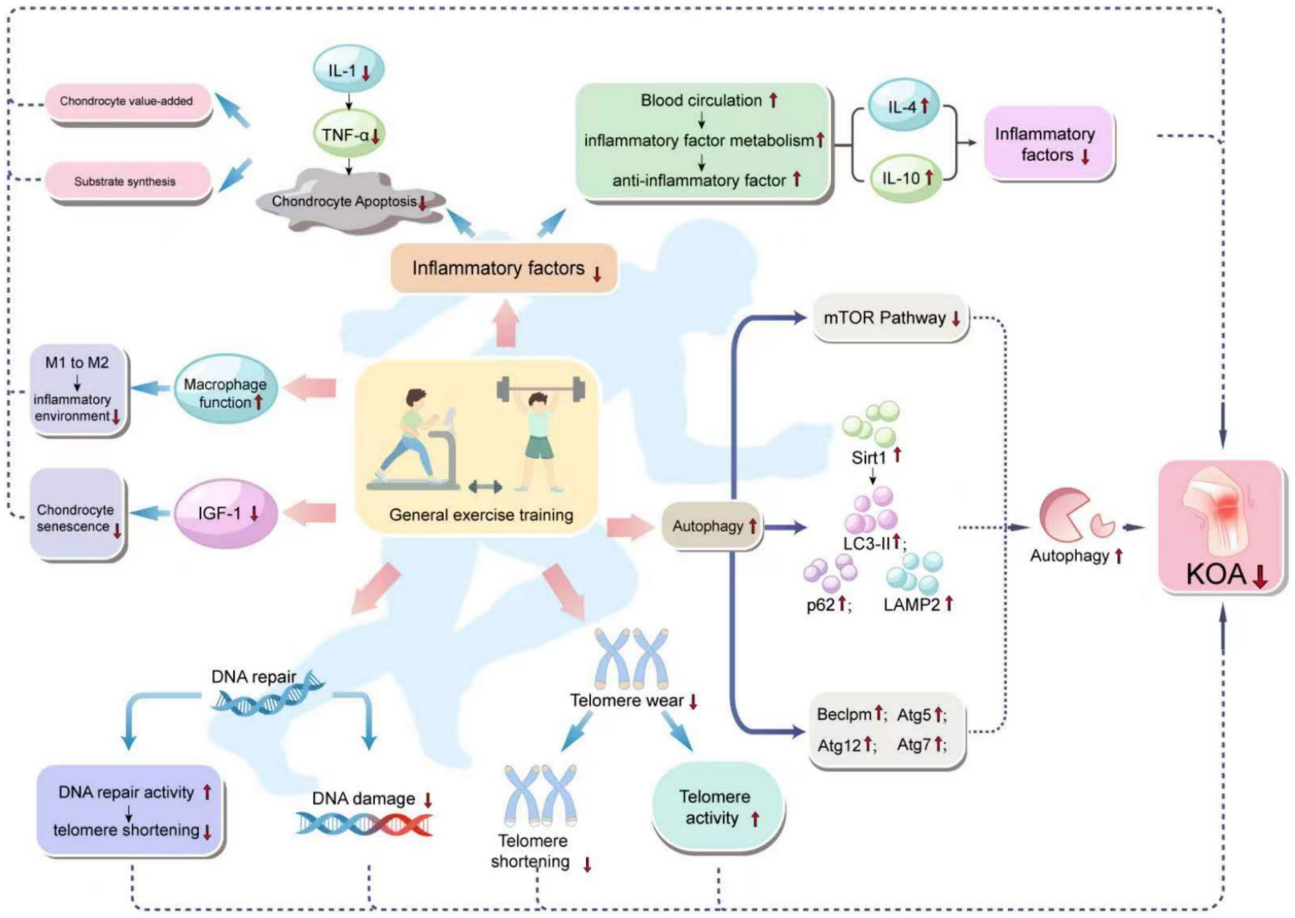
Mechanism of how general exercise training improves KOA progression through cartilage cells. General exercise training affects various biological pathways in the body, especially those related to inflammation, autophagy, DNA repair, telomere attrition, and cell function. The diagram links these effects with a reduced risk of KOA. Specifically, general exercise training enhances autophagy, DNA repair, and macrophage function, and reduces IGF-1 levels, all of which help to reduce DNA damage and telomere wear, increase telomere activity, and thus reduce cell aging and apoptosis, especially in chondrocytes. Enhanced autophagy, through the activation of the mTOR pathway and the influence on the levels of Sirt1, LC3-II, p62, and LAMP2, as well as increased expression of Beclin1, Atg5, Atg12, and Atg7, reduces chondrocyte aging and apoptosis. Improved DNA repair capacity by reducing DNA damage and telomere attrition, promotes telomere activity, which helps to delay cellular aging. The enhancement of macrophage function, shown by the shift from M1 to M2 type, helps to improve the inflammatory environment. Enhanced blood circulation, by reducing inflammatory factors and increasing anti-inflammatory factor levels, helps chondrocyte survival and function, reducing chondrocyte apoptosis. The reduction of inflammatory factors is associated with increased levels of IL-4 and IL-10, which are anti-inflammatory cytokines that help in the proliferation of chondrocytes and matrix synthesis, ultimately reducing the incidence of KOA.

Moreover, exercise training significantly impacts the improvement of systemic inflammatory conditions by enhancing the activity of regulatory T cells and reducing the ratio of inflammatory monocytes, thereby alleviating inflammation and enhancing immune function. Exercise also promotes the transformation of macrophages from the M1 to the M2 type, thereby reducing inflammation levels and, consequently, the inflammation of adipose tissue (24). Furthermore, moderate exercise training not only has a positive impact on the symptoms of KOA patients but also operates through various biological mechanisms, including improving blood circulation, enhancing the metabolism of inflammatory factors and the expression of anti-inflammatory cytokines, reducing inflammation and chondrocyte aging, inhibiting inflammation-related signaling pathways, decreasing chondrocyte apoptosis, promoting chondrocyte proliferation and matrix synthesis, thereby delaying cellular aging, repairing DNA damage, and reducing telomere attrition (3). Figure 1 illustrates how general exercise training improves the progression of KOA through chondrocyte mechanisms.

##### 3.2.1.1 Inhibition of inflammatory factors in cartilage cells

One of the characteristics of OA is synovial inflammation, and the main effector cells of the inflammatory response should be synoviocytes, KOA is no exception (17). Inflammatory factors refer to the chemicals released by cells in the body in response to external stimuli, triggering inflammation and maintaining homeostasis (25). These factors play a crucial role in the progression of KOA. A significant body of evidence indicates that the accumulation of inflammatory factors in the body leads to a chronic inflammatory microenvironment, accelerating cartilage cell senescence and exacerbating KOA (26, 27).

In the domain of human research, an experiment involving 11 healthy adult males aged 18–30 demonstrated that 30 min of vigorous cycling can enhance blood circulation and accelerate the metabolism of inflammatory factors. This study directly observed an increase in the expression of anti-inflammatory cytokines, such as interleukin 4 (IL-4) and interleukin 10 (IL-10), thereby reducing the accumulation of inflammatory factors within the body (28). Review studies further corroborate this finding, indicating that intense exercise and training can shift T-cell immunity towards an anti-inflammatory state, diminishing the inflammatory response capability of the immune system to challenges (29). Additionally, another review unveils that physical activity induces an anti-inflammatory response in certain cytokines through the PI3K/Akt and HDAC3/NF-κB pathways, a discovery based on combined research from human and animal models (30).

Studies on animal models provide further understanding of the mechanisms involved. For instance, research on an OA model in New Zealand white rabbits shows that exercise can reduce the signaling of IL-1 and tumor necrosis factor α (TNF-α) by inhibiting the JNK/NF-κB inflammatory pathway, thereby promoting the proliferation and matrix synthesis of chondrocytes (31). Research on male Wistar rats also indicates that treadmill and swimming exercises can reduce the expression of IL-1β, Caspase-3, and matrix metalloproteinase (MMP-13) in chondrocytes (32). This demonstrates how animal models elucidate specific molecular mechanisms by which exercise mitigates inflammation and promotes cartilage health. Studies on exercising horses further emphasize the universality of the impact of exercise on anti-inflammatory cytokines, showing that physical activity increases the expression of IL-6, primarily produced by skeletal muscle cells, and can induce the production of the IL-1ra receptor antagonist, stimulating the production of anti-inflammatory cytokines (33). This provides evidence on how exercise regulates inflammation across different organisms. A review focusing on the effects of exercise on IL-6 also elucidates the regulatory influence of physical activity on anti-inflammatory cytokines, with the findings derived from numerous studies involving both human and animal models (34). Furthermore, research on the Ptpn2 mouse model demonstrates that during inflammation, IL-6 can limit the expression of inflammatory cytokine genes and enhance the immune cells’ response to IL-4 (35). This further confirms the mechanisms by which exercise modulates inflammatory responses.

In summary, through direct human studies and indirect evidence from animal models, it is evident that exercise plays a significant role in regulating inflammatory factors, promoting the expression of anti-inflammatory cytokines, and mitigating the arthritic inflammation process through specific molecular pathways.

##### 3.2.1.2 Encouraging autophagy in chondrocytes

Autophagy serves as a cellular self-defense mechanism and plays a crucial role in the regulation of eukaryotic cell senescence. Research has shown that inhibiting the mechanistic target of rapamycin (mTOR) pathway can activate autophagy, suggesting a potential inhibitory effect of autophagy on cell senescence regulation (36). Specifically, autophagy can maintain the homeostasis of chondrocytes, reduce the level of joint inflammation, prevent chondrocyte death and matrix degradation, thereby improving joint symptoms, which may slow the progression of OA (37). However, with increasing age, autophagy activity decreases (38).

In the realm of animal models, through the investigation of mouse models, Huang et al. (39) discerned that physical activity can enhance autophagic capabilities, specifically manifesting in the augmented expression of sirtuin 1 (Sirt1) and the modulation of proteins associated with the autophagic process, such as LC3-II, p62, and LAMP2. Subsequent investigations, such as the work by Pinto et al. (40), similarly employed mouse models to demonstrate how physical exertion enhances the levels of autophagy-related proteins, including LC3-II, p62, and LAMP2, in addition to other autophagy-regulating proteins such as Beclin-1, Atg5, Atg12, and Atg7. This also impacts upstream signaling pathways, including AMPK, phosphorylated AMPK, and FOXO3, while concurrently downregulating the ratio of p62 to LC3-II/LC3-I, exhibiting an anti-aging effect.

Despite these studies providing animal model evidence on how exercise promotes autophagy, there currently exists a paucity of data directly derived from human studies to prove the applicability of these mechanisms to human subjects, particularly patients with KOA. Hence, the translation of these findings from animal models to human research is imperative for a deeper understanding of how exercise may ameliorate chondrocyte aging through the disturbance of the autophagic process, thereby delaying the progression of KOA.

##### 3.2.1.3 DNA repair in chondrocytes

DNA damage is identified as one of the pivotal factors contributing to the senescence of chondrocytes. In studies conducted on human chondrocytes by Copp et al. (41), it was observed that with the progression of age and OA, the burden of DNA damage escalates. Concurrently, *in vitro* research by Zhang et al. (42) unveiled the ligand-independent role of estrogen receptor-α in mitigating DNA damage-induced senescence in chondrocytes.

Concerning the benefits of exercise on DNA damage repair, both direct and indirect evidence has been provided. Studies on a cardiomyopathy rat model by Ghignatti et al. (43) demonstrated that preventative aerobic training could safeguard sympathovagal function and enhance the DNA repair capability of peripheral blood mononuclear cells. In human studies, research by Orange et al. (44) found that acute aerobic exercise could modulate DNA damage through interleukin-6, thereby reducing the proliferation of colon cancer cells. Though these studies do not directly target KOA and chondrocytes, they underscore the potential benefits of exercise in promoting DNA damage repair.

A review article by Hernández-Álvarez et al. (45) further explores the exercise-induced mechanisms of DNA protection, synthesizing a vast array of human and animal studies. Exercise intervention in aging model rats, as studied by BioMed Research International (46), was found to significantly ameliorate DNA damage, thereby benefiting chondrocyte senescence. Additionally, a meta-analysis by Tryfidou et al. (47) revealed the impact of acute exercise on DNA damage and repair in both trained and untrained individuals (including young and elderly athletes), indicating that acute exercise can activate DNA repair pathways, thus protecting immune cells. Further supporting these findings, research by Moreno-Villanueva et al. (48) observed the effects of acute exercise on DNA repair and PARP activity before and after irradiation in lymphocytes from trained and untrained individuals.

##### 3.2.1.4 Retarding telomere wear and tissue senescence

Telomeres, special chromatin structures located at the end of chromosomes, consist of telomere DNA sequences and binding proteins (49). Telomere length is closely associated with OA (50), and telomere shortening results in metabolically active cells leading to tissue aging (51), being considered as a hallmark of aging (52).

A comprehensive review of several human and animal model studies suggests that exercise can alleviate telomere shortening. Individuals engaging in physical exercise have longer telomeres compared to non-participants (53). Even daily leisure activities contribute to reducing telomere attrition (54). Exercise can also influence the activity of telomerase, with studies showing a significant increase in telomerase activity during physical exercise. A randomized controlled trial found that exercise can increase the content of telomere repeat binding factor 2 (TRF2), effectively preventing telomere shortening (55). Additionally, a study analyzing 284,479 participants from the UK Biobank found that individuals who consistently engage in physical activities have a lower aging acceleration index, indicating longer telomeres (56). Therefore, exercise plays a positive role in alleviating telomere shortening and delaying chondrocyte aging.

From the above findings, it is evident that telomere attrition and tissue aging are directly influenced by human studies and animal models. These findings not only provide important clues for understanding the underlying mechanisms but also offer valuable references for the application of these mechanisms in the treatment and prevention of OA in humans. Although further research is needed to explore the specific mechanisms involved, current information suggests that exercise appears to be a simple and effective way to delay telomere shortening and the process of chondrocyte aging.

##### 3.2.1.5 Promotion of macrophage transition

In OA, particularly in the synovium of KOA, macrophages constitute the principal immune cells, a finding corroborated by direct studies on human tissue samples (57). Recent articles reveal that macrophage functionality undergoes alterations with advancing age, specifically a decline in effector capabilities, evidenced by reduced expression of toll-like receptors (TLRs) on their surface, leading to a decreased expression of TNF-α and IL-6 post-stimulation, while production of IL-10 increases. This phenomenon has been observed in both human and animal models (58, 59). The diminished reactivity of aged macrophages to inflammatory stimuli, also grounded in research on human and animal models (58, 59), leads to an increased accumulation of inflammation-induced alternatively activated macrophages, thereby promoting angiogenesis and an enhanced production of inflammatory cytokines, which in turn accelerates the degradation of cartilage matrix and senescence of chondrocytes (60).

Moreover, macrophages can be categorized into M1 and M2 types, with M1 macrophages exerting pro-inflammatory effects, exacerbating the progression of OA, while M2 macrophages may alleviate the development of OA and promote cartilage repair. This differentiation mechanism has been observed in studies involving both human and animal models (61). Specifically, treadmill training has been found to induce changes in the ratio of M1/M2 macrophages in mouse blood, altering the synovial microenvironment. This mechanism, confirmed directly through animal experiments, promotes the transformation of M1 macrophages to M2 macrophages, thereby inhibiting the polarization of macrophage populations towards a pro-inflammatory M1 phenotype (62). These studies unveil how exercise, by facilitating macrophage transformation, ameliorates the internal inflammatory milieu, thus inhibiting chondrocyte aging and offering potential protective effects for KOA patients.

Although these investigations do not directly elucidate how exercise training impacts the M1/M2 macrophage ratio, they indeed underscore the significance of macrophage polarization in inflammatory diseases, providing a theoretical foundation for further research into how exercise training may modulate inflammation through such polarization. General physical training, as a potential intervention to affect macrophage polarization and function, warrants further exploration.

##### 3.2.1.6 Decreased expression of insulin-like growth factor 1

Insulin-like growth factor 1 (IGF-1) is a pivotal hormone implicated in metabolic/anabolic processes and mediates a multitude of benefits derived from physical exercise. This assertion is substantiated by a comprehensive review and analysis of literature sourced from MEDLINE, EMBASE, Scopus, and the Web of Science databases up to December 2015 (63). In a review by Liu et al. (64), a summary of various studies involving both human and animal models revealed that IGF-1, predominantly synthesized in the liver and present in the bloodstream, can also be produced within cartilaginous tissues. Binding of IGF-1 to its receptor facilitates a cascade of biological effects.

Specifically, research by Ashraf et al. (65) demonstrated that in rat models, IGF-1 could enhance the expression and activation of Akt in chondrocytes, thereby increasing the activity of β-galactosidase associated with cellular aging, along with the expression of aging markers p53 and p21, thus accelerating the senescence of cartilage cells. Concerning the impact of exercise on IGF-1 production, studies in rats have confirmed that exercise interventions can influence the generation of IGF-1 (66).

In human research, a meta-analysis involving breast cancer survivors indicated that exercise could decrease the levels of IGF-1 circulating in the blood (67). These findings collectively suggest that through the reduction of circulating free IGF-1 levels, exercise may contribute to the inhibition of chondrocyte senescence and the delay in the progression of KOA.

#### 3.2.2 Traditional Chinese exercise mechanisms for improving KOA: primarily based on modulating immune function

**Figure 2:**
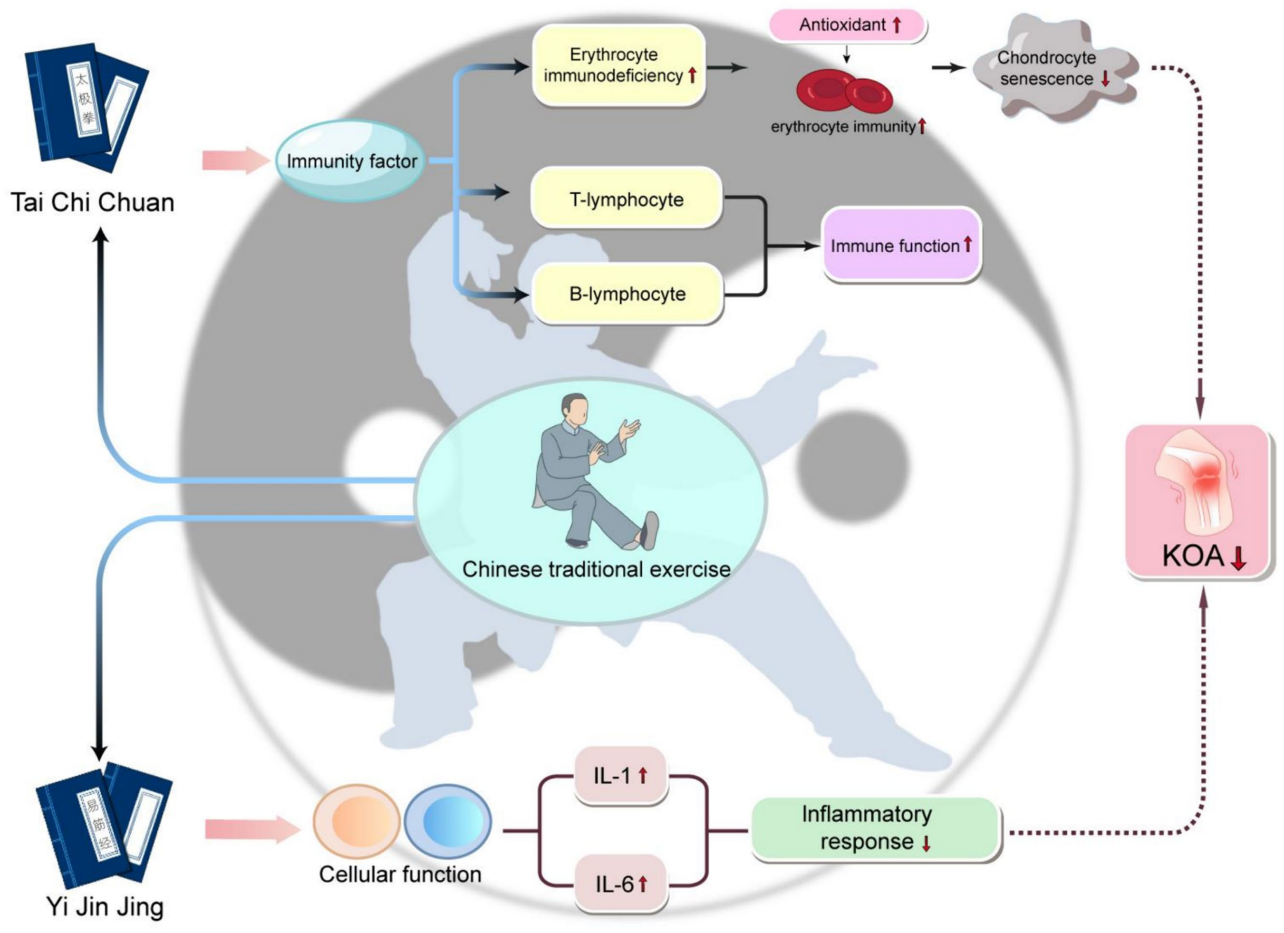
The schematic diagram of the mechanism of Traditional Chinese sports in improving KOA progression practicing traditional Chinese exercises such as Yi Jin Jing and Tai Chi can have a beneficial impact on KOA. These exercises reduce inflammatory responses by lowering cytokines like IL-1 and IL-6, which is particularly advantageous in cases where inflammation is a key issue in OA. Additionally, these exercises enhance immune factors, including red blood cell immunity, T lymphocytes, and B lymphocytes, thus strengthening overall immune function. Moreover, these exercises also increase the levels of antioxidants, further enhancing the immunity of red blood cells. This series of changes leads to a reduction in the aging of cartilage cells, ultimately slowing down the symptoms or progression of KOA.

Evidence demonstrates that traditional Chinese sports can modulate immune functions and immunological factors, thereby alleviating the inflammatory responses in the joints of patients with KOA and enhancing tissue repair. Exercises such as Yijinjing and Tai Chi have been proven to bolster the immune functions of red blood cells, T lymphocytes, and B lymphocytes in elderly women, thus mitigating local inflammation and pain (68). However, to fully comprehend the mechanisms through which traditional Chinese sports improve KOA, further research is necessary. A schematic diagram illustrating the mechanism by which Traditional Chinese Medicine sports improve the progression of KOA is shown in Figure 2.

##### 3.2.2.1 Modulating cellular function

Traditional exercises can modulate immune function to a certain extent (69), which provides valuable insights and research value for the treatment of KOA. Traditional exercises have a regulatory and modifying effect on immune factors in the body, which can alleviate inflammation reactions. It has been found that the Yi Jin Jing exercise can reduce the levels of IL-1 and IL-6 in KOA patients, thereby mitigating inflammation reactions and influencing the repair of soft tissues within the joints (70). This suggests that exercise through traditional practices may be related to immune regulation.

Owing to the unique characteristics of traditional Chinese exercises, their effects can only be discerned through human studies. A particular clinical investigation focusing on the elderly demographic has unveiled the potential impacts of traditional exercises on immunomodulation. The study reveals that older adults who participated in traditional Tai Chi/Qigong classes exhibited a certain degree of modulation in their immune response following influenza vaccination (69). This finding, derived directly from human research, provides crucial insights and a foundation for research into the therapeutic application of traditional exercises for treating KOA.

Further support is provided by a Meta-analysis examining the impact of Yijin Jing exercises on inflammation markers in KOA patients, such as IL-1 and IL-6. The analysis indicates that participation in traditional exercises like Yijin Jing significantly reduces the levels of these inflammatory markers, thereby alleviating inflammatory responses and facilitating the repair of soft tissues within the joints (71). This conclusion, also based on human research, further underscores the potential role of traditional exercises in immunomodulation, particularly in the study and treatment of human KOA patients.

##### 3.2.2.2 Regulating immune factors

Traditional Chinese exercises, such as Tai Chi and Qigong, have been implicated in the modulation of immunological factors, possessing potential roles in the context of KOA. Human studies have revealed that Tai Chi can effectively enhance the immune adhesion function of erythrocytes in the elderly, thereby improving cellular resistance to complement attack, enhancing antioxidant capabilities, and facilitating the development of erythrocyte immune functions (68). This investigation directly showcases the positive impact of Tai Chi on the immune function of the elderly. Moreover, a systematic review and meta-analysis conducted by Oh et al. (71), based on human studies, suggests that Qigong and Tai Chi can exert physiological effects on the human immune response to some extent. This comprehensive analysis, integrating data from various studies, provides a human research foundation for the potential benefits of Qigong and Tai Chi on the immune system. Furthermore, research by Vera et al. (72) and Wang et al. (73), also based on human samples, respectively found that Qigong and Tai Chi could increase the number of B lymphocytes and T lymphocytes, and potentially influence their functions. These findings underscore the direct evidence, obtained through human research, of the regulatory effects of traditional Chinese exercises on the immune system.

However, despite these studies offering valuable insights into how traditional Chinese exercises could ameliorate localized inflammation and pain through the modulation of immune cells within the body, they have not directly addressed patients with KOA. Hence, although these findings lay the groundwork for understanding the potential benefits that Tai Chi and Qigong may offer to patients with KOA, their direct applicability and mechanisms still require further elucidation through research focused on patients with KOA.

### 3.3 Effects of exercise on clinical efficacy in KOA

Following a comprehensive study of the biological mechanisms by which exercise improves KOA, the focus has now shifted towards the clinical efficacy of exercise therapy in treating KOA. Physicians specializing in sports medicine and qualified exercise rehabilitation therapists are capable of formulating such exercise prescriptions, ensuring that recipients, including elderly individuals with KOA, receive the most appropriate exercise guidance (74). This section specifically analyzes the impact of exercise training on KOA patients, highlighting key aspects such as physical function, quality of life, and pain alleviation, which are crucial for patients’ daily living and overall health. Initially, the discourse delves into how exercise training specifically enhances the physical function of KOA patients, an essential metric for assessing therapeutic outcomes. Improvements in physical function are manifested not only in joint mobility and muscle strength but also in the patients’ capability to perform daily activities. Notably, Table 1 presents the impact of conventional exercise training and Traditional Chinese Medicine exercises on the clinical efficacy for KOA.

**Table 1:** Effects of general exercise training and traditional Chinese exercise on clinical efficacy in KOA.

Aspect	Exercise Type	Details	References
Improvement of physical function	General exercise training	Squat training improves knee flexion; Balance training enhances physical function; HIIT potentially beneficial	(50–52, 75)
	Traditional Chinese exercises	Gait biomechanics analysis; Qigong training for 12 weeks improves physical function; Short-term benefits from Tai Chi; Qigong	(50, 53, 54)
Improving quality of life	General exercise training	Home exercises and physical therapy improve quality of life; Aquatic exercise reduces anxiety/depression	(55–60)
	Traditional Chinese exercise	Wuqinxi and Baduanjin improve balance and quality of life	(61, 62)
Pain relief	General exercise training	Concentric and eccentric resistance training alleviate pain	(63–68)
	Traditional Chinese exercise forms	Wuqinxi, Baduanjin, Taijiquan reduce knee pain	(68, 70–72)

#### 3.3.1 Improvement of physical function

##### 3.3.1.1 General exercise training

Restricted joint mobility is a common clinical manifestation in patients with KOA. Previous studies have demonstrated that advanced KOA patients often have reduced range of joint motion and increased disability rates (76). A study showed that KOA patients who underwent 8 weeks of squat training, including 10 min of joint mobilization and muscle stretching, and squat exercises with knee flexion at 30° and 60°, experienced an increase in knee flexion range of motion after intervention. However, the improvement in knee extension was not significant, which may be related to the proprioceptive receptors in the knee joint, particularly the muscle spindles (77). Additionally, balance training has shown significant effects in improving physical function in patients with KOA. Research has demonstrated that balance training can significantly improve patients’ balance ability and functional status, but its impact on muscle strength is not clearly evident due to limited studies (78). High-intensity interval training (HIIT) is considered a potentially beneficial exercise modality for improving symptoms and physical function in patients with KOA, including pain relief and functional enhancement (79). However, the superiority of HIIT compared to other exercise modalities still lacks clear evidence.

##### 3.3.1.2 Traditional Chinese exercises

Unbalanced changes in lower limb muscle strength may lead to changes in lower limb alignment, increasing knee instability may increase the difficulty of walking and mobility, and walking pain may worsen, resulting in differences in gait compared to healthy individuals (77). Biomechanical studies of gait have been widely used in the clinical trials of KOA, with pathological changes in internal knee moments being used as an important biomechanical parameter for assessing lower limb gait patterns in KOA patients (80).

In addition, a study by Ye et al. (81) randomized 56 patients into an intervention group and a control group. The control group maintained their normal lifestyle and did not engage in additional physical exercise, while the intervention group underwent 12 weeks of Qigong training. The results showed that the physical function of KOA patients in the intervention group significantly improved (81). Moreover, a meta-analysis showed that traditional Chinese exercises (such as Tai Chi, Eight Pieces of Brocade, Qigong, etc.) may improve physical function in KOA patients in the short term, but further studies with longer intervention periods are needed to verify this effect (82).

Overall, traditional Chinese exercises positively regulates the physiological functions of the knee joint from the perspectives of bone, tendon, and muscle, correcting lower limb alignment, improving lower limb mobility and walking ability in KOA patients.

#### 3.3.2 Improving quality of life

##### 3.3.2.1 General exercise training

Pain, restricted joint movement, and other factors can lead to a decrease in quality of life for KOA patients. Studies by Yilmaz et al. (83) and Jönsson et al. (84) have shown that both a home exercise manual provided by orthopedic doctors and guidance from physical therapists on home exercise can improve quality of life for KOA patients. These interventions were conducted over a period of 6 weeks, with daily training sessions lasting 30 to 45 min. Patients’ quality of life was evaluated using the Short Form-36 (SF-36) health survey.

In addition, KOA patients have been shown to have significant pain and anxiety/depression, which are important factors contributing to their decreased function and quality of life (85, 86). A study by Sahin et al. (87) showed that two different aquatic exercise programs applied to KOA patients, one focused solely on lower limb training and the other on upper limb and trunk exercises, resulted in improved 6-min walking test and decreased levels of anxiety and depression after 3 weeks of training. These findings are consistent with related research results (88).

##### 3.3.2.2 Traditional Chinese exercise

Under the influence of accompanying symptoms such as pain, stiffness, and limited mobility, the quality of life for KOA patients is generally lower compared to healthy individuals, and their daily activities are restricted (89). Changes in their mental state can be observed depending on the severity of the symptoms and the level of their quality of life (89). The deterioration of their mental state can exacerbate both short-term and long-term knee pain (89). The ability to regulate emotions and maintain a positive mental state are equally important for the long-term psychological well-being of the patients. A study found that after 24 weeks of practicing the Wuqinxi, balance ability and quality of life of elderly female KOA patients improved significantly and persistently (90). A systematic review and meta-analyses have indicated that practicing Baduanjin exercise has statistically significant differences in SF-36 scores compared to health education and non-steroidal anti-inflammatory drug treatments (91).

#### 3.3.3 Pain relief

##### 3.3.3.1 General exercise training

The pain experienced by KOA patients is associated with articular cartilage degeneration, activation of cytokines, and structural changes in joint innervation (92, 93). Exercise training can improve the muscular strength of the knee joint flexion and extension muscles, promote blood circulation, and better protect the knee joint cartilage, ligaments, and bones, thereby helping to alleviate pain in KOA patients (94). In a study by Vincent et al. (95), 88 KOA patients were randomly divided into concentric resistance training group, eccentric resistance training group, and control group. The control group did not receive any exercise intervention, while the concentric resistance training group and eccentric resistance training group underwent two different types of resistance training for 16 weeks, twice a week. After the intervention, the patients underwent a 6 min walk test, stair climbing test, gait analysis, and pain rating scale evaluation. The results showed that both types of resistance training were able to alleviate pain in the patients, but concentric resistance training was more effective in reducing pain and maintaining its effects compared to eccentric resistance training, which is consistent with the findings of DeVita et al. (96). Furthermore, a meta-analysis validated the effectiveness of strength training in improving pain in KOA patients (97).

##### 3.3.3.2 Traditional Chinese exercise forms

General and standardized traditional Chinese exercise forms have been demonstrated to effectively alleviate knee pain caused by KOA. Most studies utilize subjective pain scales to assess the degree of relief of pain symptoms in KOA patients following traditional exercise forms. Research has found that practicing traditional exercises such as the Wuqinxi (98), Baduanjin (99, 100), and Taijiquan (101) can significantly reduce the local pain level in the knee joint of KOA patients. Among them, the WQX group maintained or improved in all nine measures from testing to follow-up, while the control group showed significant declines in uterine pain, quadriceps strength, and knee flexor strength (98). Furthermore, Baduanjin exercise provides a safe and feasible treatment option for KOA patients, alleviating pain, stiffness, and disability, thereby contributing to enhanced quadriceps strength and aerobic capacity (99). Baduanjin is considered a viable and safe exercise choice for KOA patients (100). Similarly, Tai Chi exercise has been shown to significantly alleviate local knee pain in KOA patients (101).

## 4 Comparative analysis of general exercise training and traditional Chinese exercises in KOA management

### 4.1 Summary of current progress and long-term sustainability

This review underscores the significant non-pharmacological benefits of general sports training and traditional Chinese exercises in treating KOA, a common and serious joint disease that affects the quality of life of middle-aged and elderly people. The pathogenesis of KOA involves factors like cartilage degeneration, chronic inflammation, overload, osteophyte formation, and neurogenic inflammatory factors. Exercise has shown considerable clinical efficacy for KOA, with its improvement mechanisms primarily including the inhibition of inflammatory factors in chondrocytes, promotion of autophagy, DNA repair, retardation of telomere attrition, and macrophage transformation. Traditional Chinese exercises such as Tai Chi and Yijinjing also exhibit effects on improving KOA by regulating immune functions and mitigating symptoms. Sustaining the benefits of exercise over the long term is crucial for effective KOA management. Research indicates that the advantages gained from regular exercise, including pain relief, enhanced joint function, and improved quality of life, can persist with ongoing exercise adherence. However, maintaining long-term exercise adherence remains a challenge, with factors such as patient motivation, physical capability, and access to resources playing significant roles. To ensure the sustainability of exercise benefits, strategies such as personalized exercise programs that adapt to the changing needs of patients over time, motivational interviewing, and support from healthcare providers are essential. Integrating exercise into patients’ daily routines and leveraging technology for remote monitoring and encouragement can also enhance long-term adherence.

Literature on long-term adherence rates to exercise programs in KOA patients suggests variability, with some studies showing good sustainability and others indicating a decline over time. Key to improving these rates are regular follow-ups, patient education, and addressing barriers to exercise, such as pain and lack of motivation. Future research should focus on identifying the most effective strategies for promoting sustained exercise engagement in KOA patients to maximize the long-term health outcomes of exercise therapy. By emphasizing the importance of long-term adherence to exercise and exploring methods to enhance sustainability, we can better meet the clinical needs for effective, patient-specific, non-drug interventions in the management of KOA, encouraging further research and practice in the integration of exercise into comprehensive treatment plans.

### 4.2 Efficacy comparison

Both general exercise training and traditional Chinese exercises have shown benefits in reducing pain, improving joint function, and enhancing the quality of life in individuals with KOA. RCTs have provided evidence supporting the efficacy of both interventions. For instance, studies have demonstrated that aerobic exercise, strength training, and balance exercises not only alleviate pain but also improve mobility and daily living activities. On the other hand, traditional Chinese exercises, including Tai Chi and Qigong, has been reported to reduce knee pain and improve physical function and well-being, with specific RCTs indicating significant improvements in SF-36 scores for quality of life. However, the direct comparison in efficacy is often limited by the heterogeneity of study designs and outcome measures. These contents have been summarized in Table 2.

**Table 2:** General exercise training vs. traditional Chinese exercises in KOA management.

Aspect	General exercise training	Traditional Chinese exercises
Efficacy	Includes aerobic, strength, and balance exercises Demonstrates improvement in pain relief, joint mobility, and daily activities Evidence supported by RCTs for specific exercises	Includes Tai Chi, Qigong, and similar practices Shows benefits in reducing knee pain, enhancing physical function, and improving quality of life Specific RCTs indicate significant improvements in quality of life scores (e.g., SF-36)
Accessibility	Minimal to no equipment required, can be performed at home or in community settings Highly accessible with low to no cost involved Less dependent on cultural context for adoption	Often requires instruction from qualified practitioners, which might limit accessibility based on location and instructor availability Costs associated with professional guidance could be a barrier Cultural heritage of TCE may influence adoption and practice, especially in regions where these practices are rooted in the local culture
Patient adherence	Adherence influenced by simplicity, flexibility, and ease of integration into daily routines Motivational strategies and patient education are crucial for sustaining long-term adherence Potentially higher in settings with established support systems for physical activity	Holistic approach may foster a sense of community and personal well-being, enhancing adherence Requires continuous motivation and understanding of the practices’ holistic benefits Cultural resonance and community support can play significant roles in sustaining adherence

This table summarizes the key features and differences between general exercise training and traditional Chinese exercises in managing KOA across three main aspects: efficacy, accessibility, and patient adherence. The comparison highlights the strengths and limitations of each method, providing a basis for patients and clinicians to make informed decisions tailored to individual preferences, needs, and contexts.

### 4.3 Accessibility comparison

The accessibility of general exercise training and traditional Chinese exercises varies significantly based on resources, cost, and the need for professional guidance. General exercises may require minimal to no equipment and can be performed at home or in community settings, making them highly accessible. Conversely, traditional Chinese exercises often requires instruction from qualified practitioners, potentially limiting accessibility due to the availability of instructors and the cultural context of the exercises. The cost associated with accessing professional guidance also plays a critical role in the choice of intervention.

Social and cultural factors significantly influence the accessibility of both methods. In regions with a strong cultural heritage of traditional Chinese exercises, these exercises might be more readily embraced by the community, enhancing accessibility. Conversely, general exercise training might be more accessible in settings with established infrastructure for physical therapy and rehabilitation. These contents have been summarized in Table 2.

### 4.4 Patient adherence comparison

Adherence to exercise regimens is influenced by factors such as perceived benefits, motivation, and the physical and psychological impacts of KOA. Literature suggests varying adherence levels, with motivational strategies and patient education playing pivotal roles in enhancing adherence. While some studies indicate higher adherence to traditional Chinese exercises due to its holistic approach and community support, others suggest that the simplicity and flexibility of general exercises foster greater adherence. Strategies to increase adherence could include personalized exercise programs, regular follow-ups, and integrating exercises into daily routines (74). These contents have been summarized in Table 2.

### 4.5 Comprehensive discussion

Both general exercise training and traditional Chinese exercises present advantages and limitations. General exercise training is versatile, easily customizable, and requires minimal resources, making it suitable for a wide range of patients. Traditional Chinese exercises, while potentially limited by accessibility issues, offers holistic benefits that extend beyond physical health to include mental and emotional well-being. The choice of treatment should consider patient preferences, cultural context, and specific clinical scenarios. Future research directions could include longitudinal studies comparing the long-term effects of both methods, exploring patient preferences and barriers to adherence, and integrating modern technology to enhance accessibility and adherence.

### 4.6 Potential limitations and solutions

This review may have the following limitations: (i) impact of study design flaws: common design flaws in the included studies, such as lack of random control, insufficient implementation of blinding, and inadequate control group settings, may affect the accuracy and credibility of the results; (ii) sample size and follow-up time limitations: small sample sizes and short-term follow-ups may limit the universality of the conclusions and the certainty of long-term effects; (iii) methodological heterogeneity and potential bias: emphasizing the methodological heterogeneity of the included studies (such as the type of intervention, duration, participant characteristics, etc.) and potential bias may affect the overall conclusion regarding the effects of exercise interventions; (iv) inconsistency in result reporting: Inadequate reporting of effect sizes or statistical significance tests may lead to an overinterpretation of the results; (v) although existing evidence supports the benefits of general exercise training and traditional Chinese exercises for the management of KOA, due to the limitations discussed above, we need to interpret these findings cautiously. Possible solutions to these limitations: future research should address these potential limitations by adopting more rigorous study designs, expanding sample sizes, extending follow-up times, implementing blinding, and using more consistent outcome measurement standards.

## 5 Conclusion

For patients and clinicians, the choice between general exercise training and traditional Chinese exercises should be guided by considerations of efficacy, accessibility, patient adherence, and personal preferences. A balanced approach, potentially integrating both methods, may offer the most comprehensive benefits for managing KOA. This comparative analysis not only responds to the call for a deeper understanding of treatment options but also lays the groundwork for future research aimed at optimizing care for individuals with KOA.

## Author contributions

XD: Conceptualization, Data curation, Formal analysis, Funding acquisition, Investigation, Methodology, Project administration, Resources, Software, Supervision, Validation, Visualization, Writing – original draft, Writing – review & editing. RF: Conceptualization, Data curation, Formal analysis, Funding acquisition, Investigation, Methodology, Project administration, Resources, Software, Supervision, Validation, Visualization, Writing – original draft, Writing – review & editing. JK: Conceptualization, Data curation, Formal analysis, Funding acquisition, Investigation, Methodology, Project administration, Resources, Software, Supervision, Validation, Visualization, Writing – original draft, Writing – review & editing.
